# Erratum for “Investigation of single‐nucleotide polymorphisms in the *NR3C1a* glucocorticoid receptor gene in Cocker Spaniels with primary immune thrombocytopenia”

**DOI:** 10.1111/jvim.16517

**Published:** 2022-08-13

First published: 10 June 2022 https://onlinelibrary.wiley.com/doi/10.1111/jvim.16468

Volume 36, Issue 4

Section 4 Discussion, 6th paragraph, last sentence, margin of error calculation corrected as follows. This calculation was performed with an assumed population size of 100 using Epi Info software (https://www.cdc.gov/epiinfo/index.html).

Power calculations showed that 70 dogs would be needed to estimate the prevalence of a disorder that occurred in 20% of dogs with 5% acceptable margin of error at a 95% confidence interval.

